# Crystal structure of bis­(4-benzoyl­pyridine-κ*N*)bis­(methanol-κ*O*)bis­(thio­cyanato-κ*N*)cobalt(II)

**DOI:** 10.1107/S2056989017004765

**Published:** 2017-03-31

**Authors:** Stefan Suckert, Julia Werner, Inke Jess, Christian Näther

**Affiliations:** a Institut für Anorganische Chemie, Christian-Albrechts-Universität Kiel, Max-Eyth Str. 2, D-24118 Kiel, Germany

**Keywords:** crystal structure, discrete complex, cobalt(II) thio­cyanate, 4-benzoyl­pyridine, hydrogen bonding.

## Abstract

The crystal structure of the title compound, [Co(NCS)_2_(C_12_H_9_NO)_2_(CH_3_OH)_2_], consists of cobalt(II) cations that are octa­hedrally coordinated by two N-terminal bonding thio­cyanato anions, two methanol mol­ecules and two 4-benzoyl­pyridine ligands into discrete complexes that are located on centres of inversion. These complexes are further linked by O—H⋯O hydrogen bonding between the hy­droxy H atom of the methanol ligand and the carbonyl O atom of the 4-benzoyl­pyridine ligand of a neighboring complex into layers parallel to (101). No pronounced inter­molecular inter­actions are observed between these layers.

## Abstract 2

The crystal structure of the title compound consists of discrete octa­hedral complexes that are linked by inter­molecular O—H⋯O hydrogen bonding into layers.

## Chemical context   

The synthesis of magnetic coordination compounds is still a major topic in coordination chemistry. For example, compounds in which the metal cations are linked by small-sized anionic ligands are of special inter­est because cooperative magnetic properties can be expected (Palion-Gazda *et al.*, 2015 ▸; Massoud *et al.*, 2013 ▸). In this context, we and others have reported on a number of one- or two-dimensional thio­cyanate coordination compounds that, dependent on the nature of the metal cation and the neutral co-ligand, show different magnetic properties (Palion-Gazda *et al.*, 2015 ▸; Massoud *et al.*, 2013 ▸; Suckert *et al.*, 2016 ▸; Werner *et al.*, 2015*a* ▸,*b* ▸,*c* ▸,*d* ▸). In the majority of compounds having a chain structure, the metal cations are linked by pairs of anionic ligands, whereby the co-ligands as well as the N and the S atoms of the thio­cyanate anions are always *trans*-coordinating. Surprisingly, with 4-benzoyl­pyridine and cobalt thio­cyanate we obtained a compound in which the N-donor co-ligands are still *trans* to each other, whereas the N and the S atoms of the anionic ligands show a *cis*-arrangement (Rams *et al.*, 2017 ▸). Like many other Co chain polymers, this compound represent an anti­ferromagnetic phase of single chain magnets with magnetic properties similar to that of related cobalt compounds with an all *trans*-coordination. Later on, we accidentally obtained a further crystalline phase with 4-benzoyl­pyridine as a co-ligand by reaction in methanol. Here we report on these results.

## Structural commentary   

**Figure 1:**
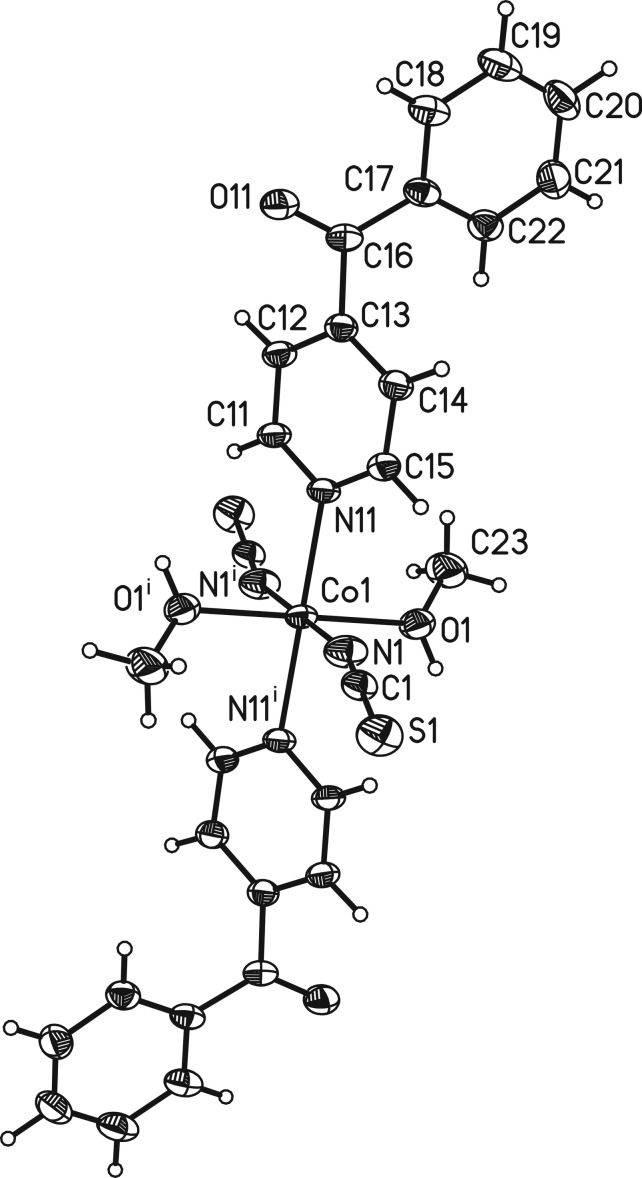
View of one discrete complex with labelling and displacement ellipsoids drawn at the 50% probability level. [Symmetry code: (i) −*x* + 1, −*y*, −*z* + 1.]

The asymmetric unit of the title compound, [Co(NCS)_2_(C_12_H_9_NO)_2_(CH_3_OH)_2_], consists of one cobalt(II) cation that is located on a center of inversion as well as of one thio­cyanate anion, one methanol mol­ecule and one neutral 4-benzoyl­pyridine ligand in general positions. The Co^II^ cation is octa­hedrally coordinated by two terminal N-bonded anionic ligands, the O atoms of two methanol mol­ecules and the N atoms of two 4-benzoyl­pyridine ligands (Fig. 1 ▸). The Co—N bond lengths to the thio­cyanate anions are significantly shorter [2.062 (2) Å] than those to the pyridine N atom of the neutral 4-benzoyl­pyridine ligand [2.1875 (18) Å]. This is expected and in agreement with bond lengths reported in the closely related structure of [Co(NCS)_2_(C_12_H_9_NO)_2_(CH_3_CN)_2_] (Suckert *et al.*, 2017 ▸) where methanol is replaced by aceto­nitrile, and also for related compounds reported in the literature (Soliman *et al.*, 2014 ▸). The 4-benzoyl­pyridine ligand is not planar, with the phenyl rings inclined by 61.34 (9)°. This value is in agreement with those retrieved from literature which vary between 40.4 and 74.8° (Escuer *et al.*, 2004 ▸).
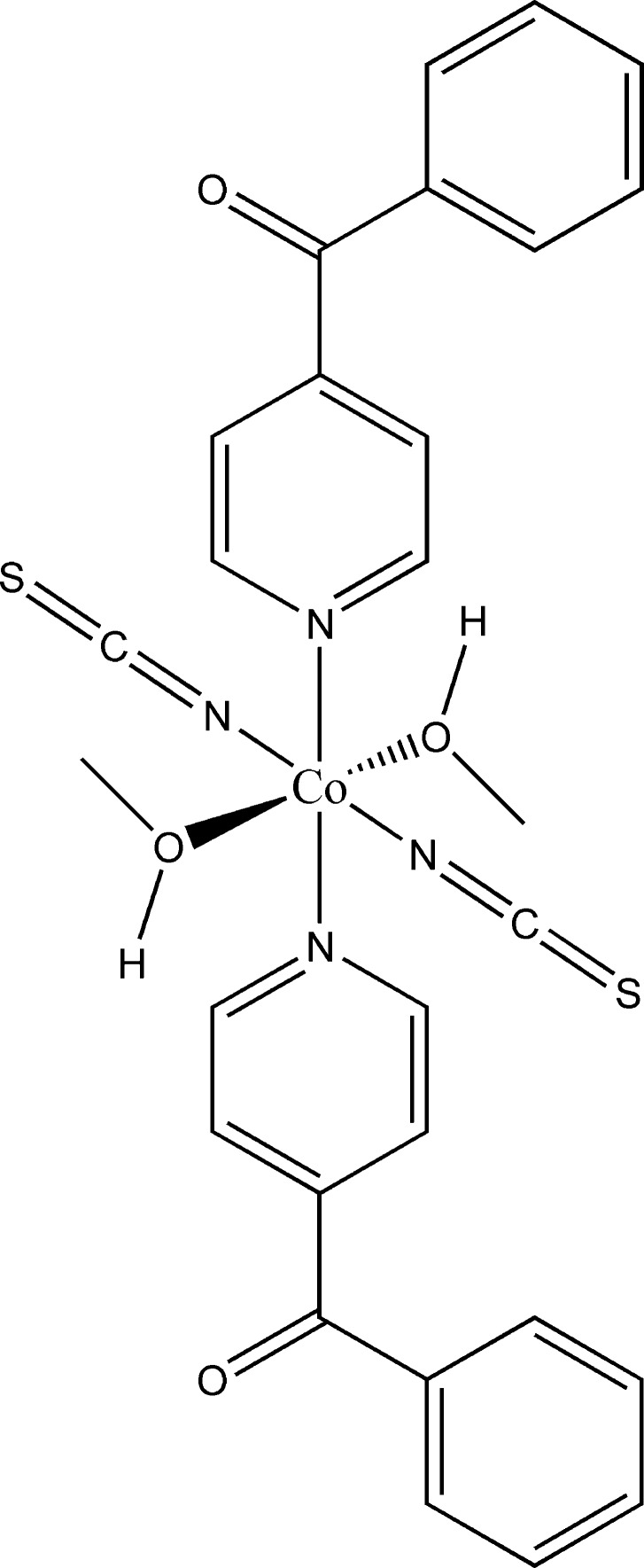


## Supra­molecular features   

**Figure 2:**
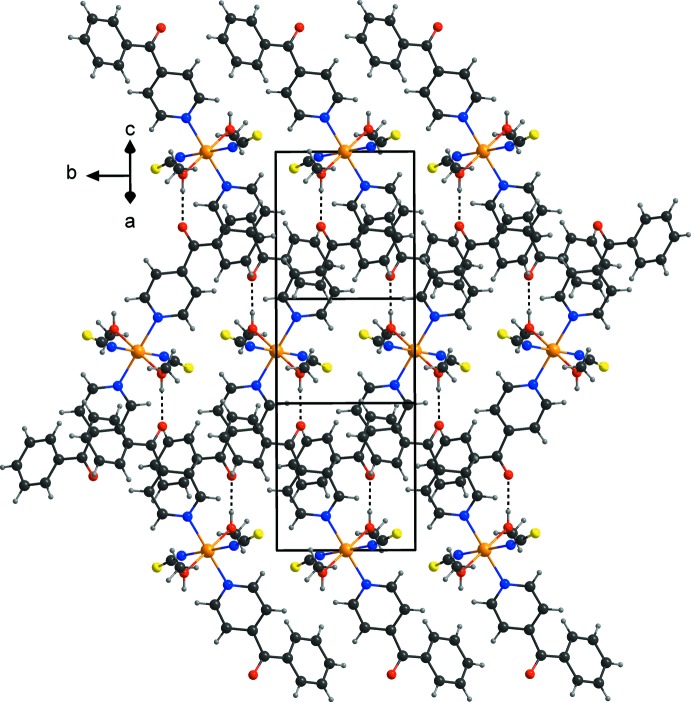
Crystal structure of the title compound in a view onto the O—H⋯O hydrogen-bonded layers (shown as dashed lines).

**Figure 3:**
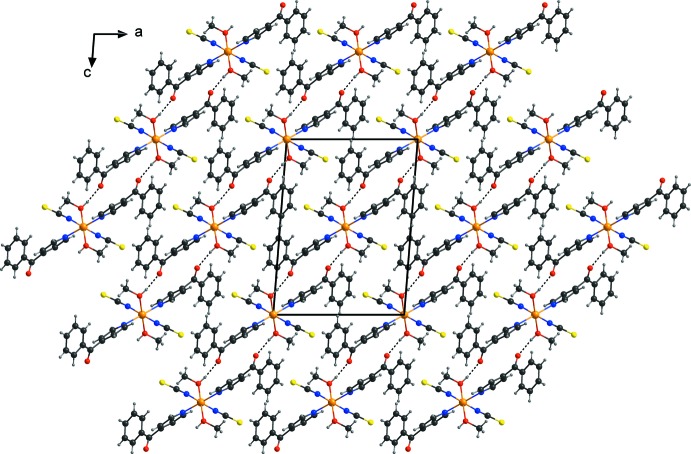
Crystal structure of the title compound in a view perpendicular to the hydrogen-bonded layers along the crystallographic *b* axis. Inter­molecular O—H⋯O hydrogen bonds are shown as dashed lines.

In the crystal structure of the title compound, the discrete complexes are linked by inter­molecular O—H⋯O hydrogen bonds between the hydroxyl H atom of the methanol mol­ecule and the carbonyl oxygen atom of a 4-benzoyl­pyridine ligand of a neighboring complex. Each of the complexes is linked to four symmetry-related complexes into layers parallel to (101) (Fig. 2 ▸ and Table 1 ▸). Between these layers no pronounced inter­molecular inter­actions are observed (Fig. 3 ▸).

## Database survey   

Altogether, there are 22 coordination compounds with 4-benzoyl­pyridine ligands compiled in the Cambridge Structure Database (Version 5.38, last update 2016, Groom *et al.*, 2016 ▸) of which three contain also thio­cyanate anions. In two of these structures Co(II) or Ni(II) cations are octa­hedrally coordinated by the N atoms of four 4-benzoyl­pyridine ligands and the N atoms of two thio­cyanate anions (Drew *et al.*, 1985 ▸; Soliman *et al.*, 2014 ▸). In the third compound, Cu(II) cations have a square-planar coordination sphere defined by two 4-benzoyl­pyridine ligands and two thio­cyanate anions (Bai *et al.*, 2011 ▸). Finally, we have reported on a compound with a one-dimensional structure, in which the Co(II) cations are linked by *μ*-1,3-bridging thio­cyanate anions (Rams *et al.*, 2017 ▸), as well as a compound very similar to the title structure in which Co^II^ cations are coordinated into discrete complexes by two thio­cyanate anions, two 4-benzoyl­pyridine ligands and two aceto­nitrile mol­ecules (Suckert *et al.*, 2017 ▸).

## Synthesis and crystallization   

Co(NCS)_2_ and 4-benzoyl­pyridine were purchased from Alfa Aesar. Crystals of the title compound suitable for single crystal X-ray diffraction were obtained by reaction of 26.3 mg Co(NCS)_2_ (0.15 mmol) with 27.5 mg 4-benzoyl­pyridine (0.15 mmol) in methanol (1.5 ml) after a few days.

## Refinement   

Crystal data, data collection and structure refinement details are summarized in Table 2 ▸. The C-bound hydrogen atoms were positioned with idealized geometry and were refined with fixed isotropic displacement parameters *U*_iso_(H) = 1.2 *U*_eq_(C) for aromatic and *U*_iso_(H) = 1.5 *U*_eq_(C) for methyl H atoms using a riding model. The methyl hydrogen atoms were allowed to rotate but not to tip. The O—H hydrogen atom was located in a difference map. Its bond length was set to the ideal value of 0.84 Å and finally, it was refined with *U*_iso_(H) = 1.5 *U*_eq_(O) using a riding model.

## Supplementary Material

Crystal structure: contains datablock(s) I. DOI: 10.1107/S2056989017004765/wm5377sup1.cif

Structure factors: contains datablock(s) I. DOI: 10.1107/S2056989017004765/wm5377Isup2.hkl

CCDC reference: 1540326

Additional supporting information:  crystallographic information; 3D view; checkCIF report

## Figures and Tables

**Table 1:** Hydrogen-bond geometry (Å, °)

*D*—H⋯*A*	*D*—H	H⋯*A*	*D*⋯*A*	*D*—H⋯*A*
O1—H1*O*1⋯O11^i^	0.84	1.92	2.752 (3)	173

Symmetry code: (i) 
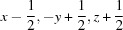
.

**Table 2:** Experimental details

Crystal data	
Chemical formula	[Co(NCS)_2_(C_12_H_9_NO)_2_(CH_4_O)_2_]
*M*_r_	605.58
Crystal system, space group	Monoclinic, *P*2_1_/*n*
Temperature (K)	200
*a*, *b*, *c* (Å)	12.0367 (10), 7.2497 (4), 16.1396 (13)
β (°)	94.404 (10)
*V* (Å^3^)	1404.22 (18)
*Z*	2
Radiation type	Mo *K*α
μ (mm^−1^)	0.80
Crystal size (mm)	0.26 × 0.20 × 0.09

Data collection	
Diffractometer	Stoe IPDS1
Absorption correction	Numerical (*X-SHAPE* and *X-RED32*; Stoe, 2008 ▸)
*T*_min_, *T*_max_	0.597, 0.901
No. of measured, independent and observed [*I* > 2σ(*I*)] reflections	13065, 3054, 2571
*R*_int_	0.094
(sin θ/λ)_max_ (Å^−1^)	0.639

Refinement	
*R*[*F* ^2^ > 2σ(*F* ^2^)], *wR*(*F* ^2^), *S*	0.052, 0.149, 1.08
No. of reflections	3054
No. of parameters	179
H-atom treatment	H-atom parameters constrained
Δρ_max_, Δρ_min_ (e Å^−3^)	0.98, −0.94

Computer programs: *X-AREA* (Stoe, 2008 ▸), *XP* in *SHELXTL* and *SHELXS97* (Sheldrick, 2008 ▸), *SHELXL2014*/7 (Sheldrick, 2015 ▸) and *DIAMOND* (Brandenburg, 1999 ▸), *publCIF* (Westrip, 2010 ▸).

## supplementary crystallographic information

### Crystal data

**Table:**

[Co(NCS)_2_(C_12_H_9_NO)_2_(CH_4_O)_2_]	*F*(000) = 626
*M**_r_* = 605.58	*D*_x_ = 1.432 Mg m^−^^3^
Monoclinic, *P*2_1_/*n*	Mo *K*α radiation, λ = 0.71073 Å
*a* = 12.0367 (10) Å	Cell parameters from 13065 reflections
*b* = 7.2497 (4) Å	θ = 2.5–27.0°
*c* = 16.1396 (13) Å	µ = 0.80 mm^−^^1^
β = 94.404 (10)°	*T* = 200 K
*V* = 1404.22 (18) Å^3^	Block, blue
*Z* = 2	0.26 × 0.20 × 0.09 mm

### Data collection

**Table:**

Stoe IPDS-1 diffractometer	2571 reflections with *I* > 2σ(*I*)
phi scans	*R*_int_ = 0.094
Absorption correction: numerical (X-SHAPE and X-RED32; Stoe, 2008)	θ_max_ = 27.0°, θ_min_ = 2.5°
*T*_min_ = 0.597, *T*_max_ = 0.901	*h* = −15→15
13065 measured reflections	*k* = −9→9
3054 independent reflections	*l* = −20→20

### Refinement

**Table:**

Refinement on *F*^2^	Hydrogen site location: mixed
Least-squares matrix: full	H-atom parameters constrained
*R*[*F*^2^ > 2σ(*F*^2^)] = 0.052	*w* = 1/[σ^2^(*F*_o_^2^) + (0.1003*P*)^2^ + 0.142*P*] where *P* = (*F*_o_^2^ + 2*F*_c_^2^)/3
*wR*(*F*^2^) = 0.149	(Δ/σ)_max_ < 0.001
*S* = 1.08	Δρ_max_ = 0.98 e Å^−^^3^
3054 reflections	Δρ_min_ = −0.94 e Å^−^^3^
179 parameters	Extinction correction: SHELXL-2014/7 (Sheldrick 2015, Fc^*^=kFc[1+0.001xFc^2^λ^3^/sin(2θ)]^-1/4^
0 restraints	Extinction coefficient: 0.026 (4)

### Special details

**Table:**

Geometry. All esds (except the esd in the dihedral angle between two l.s. planes) are estimated using the full covariance matrix. The cell esds are taken into account individually in the estimation of esds in distances, angles and torsion angles; correlations between esds in cell parameters are only used when they are defined by crystal symmetry. An approximate (isotropic) treatment of cell esds is used for estimating esds involving l.s. planes.

### Fractional atomic coordinates and isotropic or equivalent isotropic displacement parameters (Å^2^)

**Table:**

	*x*	*y*	*z*	*U*_iso_*/*U*_eq_	
Co1	0.5000	0.0000	0.5000	0.02401 (19)	
N1	0.38448 (17)	0.1893 (3)	0.45202 (14)	0.0369 (5)	
C1	0.30488 (19)	0.2635 (3)	0.42427 (14)	0.0290 (5)	
S1	0.19267 (6)	0.36720 (11)	0.38595 (5)	0.0433 (2)	
N11	0.63301 (15)	0.1453 (3)	0.44184 (12)	0.0262 (4)	
C11	0.71706 (18)	0.0512 (4)	0.41204 (15)	0.0276 (5)	
H11	0.7229	−0.0773	0.4232	0.033*	
C12	0.79607 (18)	0.1334 (3)	0.36556 (14)	0.0281 (5)	
H12	0.8528	0.0614	0.3435	0.034*	
C13	0.79087 (18)	0.3217 (3)	0.35185 (14)	0.0269 (5)	
C14	0.70540 (19)	0.4217 (4)	0.38427 (16)	0.0309 (5)	
H14	0.7002	0.5514	0.3767	0.037*	
C15	0.62774 (19)	0.3276 (3)	0.42802 (15)	0.0309 (5)	
H15	0.5685	0.3954	0.4490	0.037*	
C16	0.87501 (18)	0.4060 (3)	0.29893 (14)	0.0281 (5)	
C17	0.93531 (18)	0.5746 (4)	0.32667 (15)	0.0283 (5)	
C18	0.9973 (2)	0.6709 (4)	0.27036 (17)	0.0350 (6)	
H18	0.9958	0.6320	0.2141	0.042*	
C19	1.0602 (2)	0.8220 (4)	0.2966 (2)	0.0425 (6)	
H19	1.1008	0.8884	0.2582	0.051*	
C20	1.0640 (2)	0.8770 (4)	0.3792 (2)	0.0444 (7)	
H20	1.1081	0.9802	0.3973	0.053*	
C21	1.0038 (2)	0.7819 (4)	0.43553 (19)	0.0397 (6)	
H21	1.0072	0.8196	0.4920	0.048*	
C22	0.93860 (19)	0.6321 (4)	0.40941 (15)	0.0311 (5)	
H22	0.8962	0.5687	0.4477	0.037*	
O11	0.89175 (16)	0.3254 (3)	0.23427 (11)	0.0384 (4)	
C23	0.6398 (3)	0.2012 (6)	0.6476 (2)	0.0553 (9)	
H23A	0.6339	0.2842	0.6950	0.083*	
H23B	0.6714	0.0831	0.6673	0.083*	
H23C	0.6882	0.2568	0.6084	0.083*	
O1	0.53106 (15)	0.1711 (3)	0.60682 (11)	0.0375 (4)	
H1O1	0.4842	0.1720	0.6428	0.056*	

### Atomic displacement parameters (Å^2^)

**Table:**

	*U*^11^	*U*^22^	*U*^33^	*U*^12^	*U*^13^	*U*^23^
Co1	0.0156 (3)	0.0275 (3)	0.0295 (3)	0.00038 (14)	0.00505 (17)	0.00275 (15)
N1	0.0249 (10)	0.0380 (12)	0.0483 (12)	0.0054 (9)	0.0049 (9)	0.0121 (9)
C1	0.0262 (11)	0.0274 (12)	0.0337 (11)	−0.0023 (9)	0.0045 (9)	0.0050 (9)
S1	0.0303 (4)	0.0428 (4)	0.0555 (4)	0.0090 (3)	−0.0038 (3)	0.0089 (3)
N11	0.0190 (8)	0.0278 (10)	0.0324 (9)	−0.0027 (7)	0.0068 (7)	0.0027 (7)
C11	0.0207 (10)	0.0283 (12)	0.0350 (11)	−0.0007 (9)	0.0095 (9)	0.0030 (9)
C12	0.0206 (10)	0.0307 (12)	0.0339 (11)	0.0006 (9)	0.0079 (9)	0.0020 (9)
C13	0.0216 (10)	0.0301 (12)	0.0291 (10)	−0.0025 (9)	0.0038 (8)	0.0002 (8)
C14	0.0253 (11)	0.0259 (12)	0.0421 (13)	−0.0007 (9)	0.0077 (10)	0.0021 (9)
C15	0.0229 (10)	0.0291 (12)	0.0418 (13)	−0.0006 (9)	0.0100 (9)	−0.0024 (9)
C16	0.0193 (10)	0.0342 (13)	0.0311 (11)	−0.0006 (9)	0.0039 (8)	0.0043 (9)
C17	0.0181 (10)	0.0301 (13)	0.0368 (12)	−0.0003 (9)	0.0019 (9)	0.0060 (9)
C18	0.0258 (11)	0.0365 (14)	0.0432 (13)	−0.0037 (10)	0.0060 (10)	0.0087 (10)
C19	0.0287 (12)	0.0343 (15)	0.0655 (18)	−0.0051 (11)	0.0093 (12)	0.0109 (12)
C20	0.0266 (12)	0.0288 (14)	0.077 (2)	−0.0044 (10)	0.0012 (13)	−0.0053 (12)
C21	0.0304 (12)	0.0338 (14)	0.0545 (16)	0.0007 (10)	0.0008 (11)	−0.0081 (11)
C22	0.0224 (10)	0.0323 (13)	0.0385 (12)	0.0003 (9)	0.0023 (9)	0.0014 (9)
O11	0.0376 (10)	0.0444 (11)	0.0348 (9)	−0.0099 (8)	0.0130 (8)	−0.0012 (7)
C23	0.0412 (16)	0.079 (2)	0.0455 (16)	−0.0228 (16)	0.0037 (13)	−0.0180 (15)
O1	0.0316 (9)	0.0504 (12)	0.0313 (9)	−0.0073 (8)	0.0081 (7)	−0.0076 (7)

### Geometric parameters (Å, º)

**Table:**

Co1—N1	2.062 (2)	C16—O11	1.226 (3)
Co1—N1^i^	2.062 (2)	C16—C17	1.474 (3)
Co1—O1	2.1336 (18)	C17—C22	1.397 (3)
Co1—O1^i^	2.1336 (18)	C17—C18	1.405 (3)
Co1—N11	2.1875 (18)	C18—C19	1.379 (4)
Co1—N11^i^	2.1875 (18)	C18—H18	0.9500
N1—C1	1.159 (3)	C19—C20	1.389 (5)
C1—S1	1.626 (2)	C19—H19	0.9500
N11—C11	1.340 (3)	C20—C21	1.388 (4)
N11—C15	1.341 (3)	C20—H20	0.9500
C11—C12	1.390 (3)	C21—C22	1.386 (4)
C11—H11	0.9500	C21—H21	0.9500
C12—C13	1.383 (3)	C22—H22	0.9500
C12—H12	0.9500	C23—O1	1.435 (3)
C13—C14	1.393 (3)	C23—H23A	0.9800
C13—C16	1.504 (3)	C23—H23B	0.9800
C14—C15	1.393 (3)	C23—H23C	0.9800
C14—H14	0.9500	O1—H1O1	0.8400
C15—H15	0.9500		
			
N1—Co1—N1^i^	180.00 (10)	N11—C15—H15	118.6
N1—Co1—O1	89.30 (9)	C14—C15—H15	118.6
N1^i^—Co1—O1	90.70 (9)	O11—C16—C17	123.0 (2)
N1—Co1—O1^i^	90.70 (9)	O11—C16—C13	116.8 (2)
N1^i^—Co1—O1^i^	89.30 (9)	C17—C16—C13	120.1 (2)
O1—Co1—O1^i^	180.0	C22—C17—C18	119.5 (2)
N1—Co1—N11	90.75 (8)	C22—C17—C16	121.0 (2)
N1^i^—Co1—N11	89.25 (8)	C18—C17—C16	119.3 (2)
O1—Co1—N11	88.74 (7)	C19—C18—C17	120.1 (3)
O1^i^—Co1—N11	91.26 (7)	C19—C18—H18	119.9
N1—Co1—N11^i^	89.25 (8)	C17—C18—H18	119.9
N1^i^—Co1—N11^i^	90.75 (8)	C18—C19—C20	120.0 (3)
O1—Co1—N11^i^	91.26 (7)	C18—C19—H19	120.0
O1^i^—Co1—N11^i^	88.74 (7)	C20—C19—H19	120.0
N11—Co1—N11^i^	180.0	C21—C20—C19	120.4 (3)
C1—N1—Co1	165.5 (2)	C21—C20—H20	119.8
N1—C1—S1	179.5 (2)	C19—C20—H20	119.8
C11—N11—C15	118.02 (19)	C22—C21—C20	120.1 (3)
C11—N11—Co1	120.42 (16)	C22—C21—H21	120.0
C15—N11—Co1	121.28 (15)	C20—C21—H21	120.0
N11—C11—C12	122.8 (2)	C21—C22—C17	119.9 (2)
N11—C11—H11	118.6	C21—C22—H22	120.0
C12—C11—H11	118.6	C17—C22—H22	120.0
C13—C12—C11	119.0 (2)	O1—C23—H23A	109.5
C13—C12—H12	120.5	O1—C23—H23B	109.5
C11—C12—H12	120.5	H23A—C23—H23B	109.5
C12—C13—C14	118.6 (2)	O1—C23—H23C	109.5
C12—C13—C16	117.9 (2)	H23A—C23—H23C	109.5
C14—C13—C16	123.4 (2)	H23B—C23—H23C	109.5
C15—C14—C13	118.6 (2)	C23—O1—Co1	123.76 (18)
C15—C14—H14	120.7	C23—O1—H1O1	108.6
C13—C14—H14	120.7	Co1—O1—H1O1	118.5
N11—C15—C14	122.8 (2)		

Symmetry code: (i) −*x*+1, −*y*, −*z*+1.

### Hydrogen-bond geometry (Å, º)

**Table:**

*D*—H···*A*	*D*—H	H···*A*	*D*···*A*	*D*—H···*A*
O1—H1*O*1···O11^ii^	0.84	1.92	2.752 (3)	173

Symmetry code: (ii) *x*−1/2, −*y*+1/2, *z*+1/2.
